# Associations between circulating IgG antibodies to Apolipoprotein B_100_-derived peptide antigens and acute coronary syndrome in a Chinese Han population

**DOI:** 10.1042/BSR20180450

**Published:** 2018-11-07

**Authors:** Weina Hu, Xueying Zhang, Yunan Han, Yong Wang, Mingming Lei, Ian L. Megson, Jun Wei, Yuanzhe Jin

**Affiliations:** 1 Department of Cardiovascular Medicine, The Fourth Affiliated Hospital of China Medical University, No. 4 Chongshan East Road, Shenyang 110032, China; 2 Division of Public Health Sciences, Department of Surgery, Washington University School of Medicine, St. Louis, MO 63110, U.S.A.; 3 Department of Diabetes and Cardiovascular Science, University of the Highlands and Islands, Centre for Health Science, Inverness IV2 3JH, U.K.

**Keywords:** acute coronary syndrome, ApoB100, antibodies, atherosclerosis

## Abstract

**Objectives:** Acute coronary syndrome (ACS) is the major cause of mortality worldwide and caused mainly by atherosclerosis of coronary arteries. Apolipoprotein B100 (ApoB100) is a major component of low-density lipoprotein (LDL) and its oxidation can trigger inflammation in vascular endothelial cells leading to atherosclerosis. The association between antibodies to ApoB100-derived antigens and atherosclerotic diseases has been studied in recent years, but the findings appear to be controversial. The present study developed an ELISA in-house with ApoB100-derived peptide antigens to circulating anti-ApoB100 IgG antibodies in patients with ACS. **Methods:** Fifteen ApoB100-derived peptide antigens (Ag1–Ag15) were designed to develop an in-house ELISA for the detection of circulating anti-ApoB100 IgG levels in 350 patients with ACS and 201 control subjects amongst a Chinese population. Binary logistic regression was applied to examine the differences in anti-ApoB IgG levels between the patient group and the control group with adjustment for a number of confounding factors; the correlation between anti-ApoB100 IgG levels and clinical characteristics was also tested. **Results:** Patients with ACS had significantly higher levels of plasma IgG for Ag1 (adjusted *P*<0.001) and Ag10 antigens (adjusted *P*<0.001). There was no significant increase in the levels of IgG to the other 13 antigens in these ACS patients. In the control group, anti-Ag10 IgG levels were positively correlated with age, high-density lipoprotein (HDL), and ApoA levels (*P*≤0.001 for all) and negatively correlated with blood triglyceride (TG) (*P*=0.008); in the patient group, anti-Ag10 IgG levels were positively correlated with LDL (*P*=0.003), and negatively correlated with ApoA (*P*=0.048) and systolic blood pressure (SBP) (*P*=0.036). The area under ROC (receiver operator characteristic) curve (AUC) was 0.612 (95% confidence interval (CI): 0.560–0.664; *P*<0.001) in anti-Ag1 IgG assay and 0.621 (95% CI: 0.569–0.672; *P*<0.001) in anti-Ag10 IgG assay. **Conclusion:** Circulating IgG for ApoB100-derived peptide antigens may be a useful biomarker of ACS, although anti-ApoB IgG levels were not associated with the coronary artery plaque burden characterized by the coronary Gensini score.

## Introduction

Acute coronary syndrome (ACS) is currently a major cause of mortality worldwide [1]. The prevalence of cardiovascular diseases (CADs) in China is still rising based on a recent report [2]. ACS has been stratified into ST-elevation myocardial infarction (STEMI) and non-ST elevation ACS (NSTEACS); the latter is further classified into non-STEMI (NSTEMI) and unstable angina (UA) according to cardiac biomarkers of necrosis, such as cardiac troponin [3,4].

Atherosclerosis of coronary arteries is the main cause of ACS, which is associated with chronic inflammatory disorders [5]. Inflammatory responses could be activated by circulated low-density lipoprotein (LDL) and/or oxidized LDL (oxLDL), finally contributing to the formation of coronary artery atherosclerosis [6–8]. OxLDL and its residues have a high immunogenicity and may stimulate B cells to secrete specific antibodies against ApoB-derived epitopes [9,10]. Circulating IgG/IgM antibodies against oxLDL have been detected in atherosclerotic patients [11–13], and their association with atherosclerotic disease suggests that anti-oxLDL antibodies could serve as a biomarker of lipid peroxidation [14]. However, the roles (protective or pathogenic) of anti-oxLDL antibodies in the occurrence and progression of atherosclerosis remain in a debate [6,12,15–23]. The inconsistent findings across studies could be attributed to a few factors, such as the standardization of the antigen, the use of different antibodies like IgG or IgM antibodies against oxLDL in different studies, and the discrepancy in diagnostic criteria.

During the oxidation of LDL particles, new epitopes are formed continuously, which may depend on the different compositions of LDL particles isolated from individuals, resulting in a poorly defined antigen. Since native LDL may undergo oxidative modification that permits its uptake by antigen-presenting cells without altering the amino acid side chains of ApoB [24], detection of the antibodies against specific Apolipoprotein B100 (ApoB100)-derived peptides antigens might overcome this problem [25].

In our previous study, we designed eight human ApoB100-derived peptide antigens (hAgs), based on the computational prediction of HLA-II restricted epitopes [26,27]. We found that the levels of circulating IgG antibody for one of these eight hAgs were significantly increased in both AMI and NSTEACS patients amongst a Chinese Han population [26,27]. In fact, both aldehyde-modified and native ApoB peptides specific to oxLDL can be targetted by the immune system [28]. The antigens presented by MHC class II (MHC-II) molecules generally comprise 13–17 amino acids that can minimize the chance of cross-reactivity.

In the present study, we expanded previous work on the detection of circulating anti-ApoB100 IgG antibodies in ACS patients with more native ApoB100-derived peptide antigens (Ag1–Ag15) and a larger sample size.

## Materials and methods

### Patients and controls

The present study included 350 patients diagnosed with ACS between January 2012 and January 2014 at the Department of Cardiovascular Medicine, The Fourth Affiliated Hospital of China Medical University, Shenyang, China, of whom 130 had NSTEACS (include both UA and NSTEMI) and 220 had STEMI. All diagnoses were made based on 2014 AHA/ACC guidelines for the management of NSTEACS [29] and Third Universal Definition of Myocardial Infarction [30], with confirmation by coronary angiography. Venous blood samples were collected from each patient within 24 h after the onset of ACS and stored in a freezer at −70°C for subsequent analysis. Two hundred and one control subjects, well-matched for age and sex, were recruited from local communities during the same period. Clinical interviews and biomarker detection were applied to exclude those with a history of the CAD. All blood samples were taken after overnight fasting and plasma was separated and stored at −70°C until use. Exclusion criteria included history of malignancies, rheumatoid arthritis, and connective tissue diseases, organ transplantation and long-term use of immunosuppressive medication. The clinical characteristics of these subjects are given in Table 1.

**Table 1:** Clinical characteristics of patients with ACS and control subjects

Characteristics	Overall population (*n*=551)	ACS (*n*=350)	Control (*n*=201)	*P*-value
**Baseline characteristics**				
Age (years)	60.2 ± 9.1	60.7 ± 10.7	59.4 ± 5.4	0.113
Male, *n* (%)	390 (71%)	254 (73%)	136 (68%)	0.243
BMI*	24.9 ± 3.5	24.8 ± 3.7	25.3 ± 3.1	0.120
**Clinical diagnostics**				
STEMI, *n* (%)		226 (65%)		-
NSTEACS, *n* (%)		124 (35%)		-
**Risk factors**				
Smoking, *n* (%)	260 (49%)	193 (55%)	67 (33%)	<0.001
Hypertension, *n* (%)	242 (44%)	196 (56%)	46 (23%)	<0.001
Diabetes mellitus, *n* (%)	127 (23%)	97 (28%)	30 (15%)	0.001
Obesity, *n* (%)	101 (18%)	63 (18%)	38 (19%)	0.712
Dyslipidemia, *n* (%)	313 (59%)	229 (65%)	84 (47%)	<0.001
Family history	160 (30)	103 (29)	57(32)	0.567
**Hemodynamic parameters**				
SBP (mmHg)	131.0 ± 20.9	131.0 ± 22.2	130.9 ± 18.1	0.978
DBP (mmHg)	78.4 ± 12.9	78.5 ± 13.8	78.4 ± 10.8	0.933
Heart rate (ppm)	73.7 ± 13.9	72.5 ± 14.7	76.2 ± 11.6	0.003
**Laboratory data**				
Cholesterol (mM)	4.77 ± 1.17	4.50 ± 1.17	5.27 ± 0.97	<0.001
Triglyceride (mM)	1.84 ± 1.83	1.81 ± 1.78	1.91 ± 1.92	0.521
HDL (mM)	1.11 ± 0.32	1.02 ± 0.27	1.29 ± 0.33	<0.001
LDL (mM)	3.02 ± 0.91	2.96 ± 0.96	3.13 ± 0.81	0.057
ApoA (mM)	1.20 ± 0.22	0.94 ± 0.17	1.34 ± 0.22	<0.001
ApoB (mM)	0.88 ± 0.20	0.75 ± 0.22	0.91 ± 0.18	0.015

Abbreviations: DBP, diastolic blood pressure; HDL, high-density lipoprotein; SBP, systolic blood pressure.

* Body mass index (BMI) was calculated as weight (kg) at each age divided by attained height (m^2^) squared.

### Patient and public involvement

All subjects were of Chinese Han origin and gave informed written consent to participate in the present study, as approved by the Ethics Committee of the Fourth Affiliated Hospital of China Medical University (number EC-2013-044).

### Diagnosis and definition

All patients and controls were investigated in person by a cardiologist. Traditional risk factors for CAD were tabulated. Smoking was defined as current or a history of smoking. Based on WHO diagnostic criteria, hypertension (HTN) was defined as systolic blood pressure (SBP) ≥ 140 mmHg or diastolic blood pressure (DBP) ≥ 90 mmHg or a history of HTN; Diabetes (DM) as fasting blood glucose ≥ 7.0 mmol/l or a history of DM; dyslipidemia as follows: total cholesterol > 6.2 mmol/l or triglyceride (TG) > 2.3  mmol/l or LDL > 3.7  mmol/l or high-density lipoprotein (HDL) < 1.04  mmol/l or on cholesterol-lowering medication; and obesity as BMI ≥ 30. The family history of CAD was considered to be positive in those individuals whose first-degree relatives had presented prematurely (<55 years in men and <65 years in women) with, or died from CADs.

Two experienced interventional cardiologists who were completely blind to the study assessed the coronary angiograms of patients. The severity of coronary atherosclerosis was then determined by the Gensini score, based on the degree of coronary luminal narrowing and its geographical distribution [31].

### Autoantibody testing

Fifteen peptide antigens (hAgs) derived from native human ApoB were designed based on the computational prediction of HLA-II restricted epitopes [32] and on the epitope information for human disease in the Immune Epitope Database (http://www.iedb.org/). These 15 hAgs were all linear antigens with 25–30 amino acid residues that were applied to develop an ELISA in-house for detection of circulating anti-ApoB100 IgG levels based on the method described in our previous studies [44,45]. The sequences of these 15 antigens are given in Table 2. Each sample was tested in duplicate. To reduce the interference from a nonspecific signal produced by passive absorption of various IgG antibodies in blood to the surface of 96-well microplate, a specific binding index (SBI) was used to express the antibody levels in plasma. SBI was calculated as follows: SBI = (ODhAg − ODNC)/(ODcAg − ODNC), where OD is optical density and NC is negative control tested with assay buffer.

**Table 2:** Apo-B derived linear peptide antigens used in the study

Antigen	Sequence of antigen	Position
**Ag1**	H-DRFKPIRTGISPLALIKGMTRPLSTLIS-OH	213–240
**Ag2**	H-LQWLKRVHANPLLIDVVTYLVALIPEPS-OH	395–422
**Ag3**	H-TFLDDASPGDKRLAAYLMLMRSPSQA-OH	547–572
**Ag4**	H-TVMDFRKFSRNYQLYKSVSLPSLDP-OH	626–650
**Ag5**	H-TFKYNRQSMTLSSEVQIPDFDVDLGTILR-OH	1050–1078
**Ag6**	H-KIKGVISIPRLQAEARSEILAHWSPAKLL-OH	1119–1147
**Ag7**	H-DMTFRHVGSKLIVAMSSWLQKASGSLPY-OH	1220–1247
**Ag8**	H-ENVQRNLKHINIDQFVRKYRAALGKLP-OH	2093–2119
**Ag9**	H-SFKTFIEDVNKFLDMLIKKLKSFDYH-OH	2408–2433
**Ag10**	H-EFTILNTFHIPSFTIDFVEMKVKIIRTI-OH	2649–2676
**Ag11**	H-TFGKLYSILKIQSPLFTLDANADI-OH	2754–2777
**Ag12**	H-AHLNGKVIGTLKNSLFFSAQPFEIT-OH	3023–3047
**Ag13**	H-YKLEGTTRLTRKRGLKLATALSLSN-OH	3379–3403
**Ag14**	H-SCKLDFREIQIYKKLRTSSFALNLPT-OH	3760–3785
**Ag15**	H-FLIYITELLKKLQSTTVMNPYMKLAPGELT-OH	4531–4560
**cAg**	H-HAQLEGRLHDLPGCPREVQRGFAATLVTN-OH	-

Abbreviations: Ag, antigen; cAg, control antigen, which was derived from maize protein.

### Data analysis

Antibody testing data were expressed as mean ± S.D. in SBI. Student’s *t* test was initially applied to detect the differences in SBI between the patient and the control groups. Binary logistic regression was used to analyze the differences in circulating IgG to an antigen of interest between the patient group and the control group, with adjustment for multiple potential confounders listed in Table 1. One-way ANOVA, with Bonferroni *post-hoc* test, was applied to compare the differences in anti-ApoB100 antibody levels between subgroups of ACS patients. Spearman correlation analysis was used to test the correlation between plasma anti-ApoB100 IgG levels and other clinical characteristics. Receiver operator characteristic (ROC) curves were constructed to assess the predictive accuracy of candidate antibodies for ACS and the area under the ROC curve (AUC) was calculated. Because 15 antigens were tested in the present study, the significance level was set at *P*≤0.003 (0.05/15). The sensitivity of the ELISA antibody test was determined against a specificity of ≥90%.

### Data sharing statement

The authors are willing to share the data associated with this work

## Results

There was no significant difference in sex, age, body weight, family history, blood triglyceride, and LDL levels between ACS patients and control subjects. Compared with control subjects, however, patients with ACS had a higher prevalence of smoking, HTN, diabetes, and dyslipidemia, and lower heart rates, serum cholesterol, HDL, ApoA, and ApoB levels (Table 1).

Plasma levels of IgG against Ag1 and Ag10 were significantly higher in the patient group than the control group after adjustment for age, sex, heart rates, smoking, HTN, diabetes, cholesterol, HDL, LDL, ApoA, and ApoB (*P*<0.001); the other 13 antigens were not significantly different between patients with ACS and controls (Table 3). As shown in Table 4, one-way ANOVA showed a significant increase in anti-Ag1 and anti-Ag10 IgG levels in patients with either NSTEACS or STEMI as compared with control subjects (F = 9.149, df = 2548, *P*<0.001 for anti-Ag1 IgG, and F = 7.348, df = 2548, *P*=0.001 for anti-Ag10 IgG), but there was no significant difference observed between patients with USTEACS and those with STEMI. The Gensini score of coronary lesions was significantly higher in patients with STEMI than those with NSTEACS (*P*=0.01).

**Table 3:** The levels of circulating anti-Apo-B IgG antibodies in ACS circulation

Antigen	ACS patients (SBI) (*n*=350)	Control (SBI) (*n*=201)	*P-*value
**Ag1**	0.798 ± 0.148	0.744 ± 0.140	<0.001^*^
**Ag2**	2.195 ± 0.672	2.087 ± 0.691	0.080
**Ag3**	0.954 ± 0.223	0.958 ± 0.218	0.852
**Ag4**	0.926 ± 0.191	0.889 ± 0.133	0.017
**Ag5**	1.049 ± 0.115	1.056 ± 0.142	0.688
**Ag6**	0.890 ± 0.176	0.926 ± 0.215	0.058
**Ag7**	0.701 ± 0.158	0.686 ± 0.167	0.356
**Ag8**	0.894 ± 0.191	0.858 ± 0.193	0.039
**Ag9**	0.689 ± 0.161	0.696 ± 0.148	0.582
**Ag10**	0.942 ± 0.427	0.794 ± 0.392	<0.001^†^
**Ag11**	1.015 ± 0.192	0.973 ± 0.168	0.012
**Ag12**	0.878 ± 0.134	0.851 ± 0.135	0.028
**Ag13**	1.024 ± 0.207	1.044 ± 0.257	0.337
**Ag14**	0.915 ± 0.157	0.921 ± 0.174	0.714
**Ag15**	0.693 ± 0.166	0.696 ± 0.124	0.893

* *P*<0.001. ^†^*P*<0.001 after adjustment for age, gender, smoking, HTN, diabetes, BMI, familial history, cholesterol, HDL, LDL, ApoA, ApoB, and medication.

**Table 4:** The levels of anti-Apo-B IgG antibodies of Ag1 and Ag10 in the subgroup of ACS

IgG antibodies	NSTEACS (*n*=130) (SBI)	STEMI (*n*=220) (SBI)	Control (*n*=201) (SBI)	F (df)	*P*-value
Anti-Ag1	0.810 ± 0.155^*^	0.792 ± 0.144^†^	0.744 ± 0.139	9.149 (2548)	<0.001
Anti-Ag10	0.941 ± 0.444^‡^	0.942 ± 0.419^§^	0.794 ± 0.392	11.188 (2548) (2548)	0.001
Gensini score	42.54 ± 34.60	51.86 ± 27.72	-	3.642	0.01

Values are presented as mean ± S.D. in SBI. Abbreviation: STEMI, ST-segment elevation myocardial infarction.

* *P*<0.001, compared with the control group. ^†^*P*=0.001, compared with the control group.

‡ *P*=0.003, compared with the control group. ^§^*P*<0.001, compared with the control group.

As shown in Table 5, Spearman correlation analysis demonstrated that anti-Ag10 IgG antibody was positively correlated with age (*P*<0.001), HDL (*P*=0.003) and plasma ApoA concentrations (*P*<0.001), and negatively correlated with TG levels (*P*=0.007) in control subjects; anti-Ag10 IgG antibody was positively correlated with LDL in patients with ACS (*P*=0.003). No correlation was observed between IgG antibody to Ag1 and clinical characteristics (Supplementary Table S1).

**Table 5:** Spearman correlation between levels of anti-Ag10 IgG and clinical characteristics

	ACS patients			Control		
	*N*	r	*P*-value	*N*	r	*P*-value
**Age**	341	−0.013	0.805	196	0.359	<0.001
**SBP**	341	−0.095	0.080	196	0.086	0.280
**DBP**	341	−0.090	0.099	196	0.069	0.383
**Heart rate**	341	0.013	0.816	196	0.023	0.768
**BMI**	338	−0.039	0.518	194	−0.147	0.063
**Glucose**	340	0.050	0.365	195	0.153	0.052
**CHOL**	341	0.103	0.065	196	0.095	0.232
**TG**	341	−0.003	0.963	196	-0.212	0.007
**HDL**	341	0.022	0.697	196	0.233	0.003
**LDL**	341	0.166	0.003	196	0.056	0.480
**Apo-A**	341	0.022	0.721	194	0.310	<0.001
**Apo-B**	341	0.109	0.078	194	0.036	0.651
**Gensini score**	341	0.005	0.931			

Abbreviations: ApoA, apolipoprotein A; ApoB, apolipoprotein B; BMI, body mass index.

**Figure 1:**
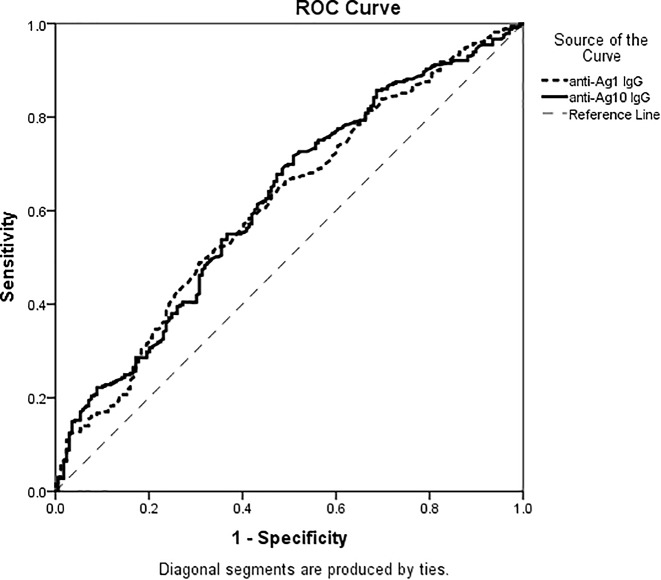
ROC curve of anti-Ag1 IgG and anti-Ag10 IgG antibodies for the predicting of ACS The AUC of anti-Ag1 IgG was 0.612 (95% CI: 0.560-0.664) (*P*<0.001 compared with AUC = 0.5) and the AUC of anti-Ag10 IgG was 0.621 (95% CI: 0.569–0.672) (*P*<0.001 compared with AUC = 0.5).

ROC curve analysis showed that AUC for anti-Ag1 IgG was 0.612 (95% confidence interval (CI): 0.560–0.664; *P*<0.001) and that for anti-Ag10 IgG was 0.621 (95% CI: 0.569–0.672; *P*<0.001), respectively (Figure 1).

Analysis of a quality control sample that was pooled with plasma randomly taken from >30 individual donors showed an inter-assay deviation of <20% for all 15 individual antibody tests (Supplementary Table S2).

## Discussion

The present study revealed that the levels of circulating IgG antibodies against two linear antigens (Ag1 and Ag10) derived from native ApoB100 protein were significantly higher in ACS patients compared with control subjects in a Chinese Han population. However, the increased anti-ApoB IgG levels were not associated with the coronary artery plaque burden characterized by the coronary Gensini score. Combined with ROC curve analysis, anti-ApoB100 IgG antibodies could potentially have a diagnostic value of ACS, independent of other traditional risk factors.

Fredrikson et al. [25] developed an ELISA based on a library of 20-mer polypeptides covering the full-length ApoB100 sequence, in order to identify antigenic peptides recognized by autoantibodies present in human plasma. They found that a number of epitopes within the ApoB100 component of oxLDL provoked an immune response in humans. However, whether this immune response was adaptive (protective) or maladaptive (causal) in atherosclerosis remains unknown. Both innate immunity and adaptive immunity play a significant role in the development and progression of atherosclerosis [8,33,34]. There are two most important autoantigens that may trigger adaptive immune responses, oxLDL and ApoB100 [25,35]. Antibodies against oxLDL and ApoB100 have been studied for several decades [36], but the relations between these antibodies and atherosclerotic diseases have not been established [34,37,38]. Recent studies have revealed a protective role of autoantibodies against ApoB100-derived peptides p45 or p210 in the development of CVD, diabetes, and systemic lupus erythematosus [39–46]. In our previous studies [26,27], however, we observed a positive association of plasma IgG antibody against native ApoB100-derived linear peptide antigens with USTEACS and AMI in a Chinese population. In assumption, increased oxLDL levels in the circulation and vascular endothelial cells could be captured by macrophages and dendritic cells, which are professional antigen-presenting cells, and as a result, MHC II-restricted ApoB100-derived peptide epitopes are presented to CD4^+^ T cells, leading to inflammatory immune responses that in turn contribute to the development of atherosclerosis.

In the baseline characteristics, the prevalence of traditional risk factors for ACS, such as smoking, HTN, diabetes, and dyslipidemia, was unsurprisingly significantly higher in the patients with ACS than control subjects [47]. The plasma concentrations of HDL and ApoA were significantly lower in the patient group than the control group (Table 1), consistent with the notion that HDL is protective against ACS. Heart rate, serum cholesterol, and ApoB100 levels were also significantly lower in ACS patients than control subjects (Table 1), which may be related either to the characteristics of the disease itself or to the use of β-blockers, aspirin and statins for HTN and hypercholesterolemia, respectively. Given that almost every patient was prescribed statins, aspirin, and β-blockers for the treatment of ACS, we still failed to find the correlation between these drugs and anti-ApoB100 antibodies in control subjects (Supplementary Table S3).

The present study revealed that only anti-Ag1 and anti-Ag10 IgG levels were significantly increased in the patients with ACS compared with controls (Table 3). Failure to show an increase in the levels of IgG antibodies against other ApoB100-derived antigens suggests that Ag1 and Ag10 may be more antigenic for the immune system than the other ApoB100-derived peptides, suggesting that epitope mapping is particularly useful for the development of an epitope-specific antibody test. The patients with either NSTEACS or STEMI had significantly higher anti-Ag1 and anti-Ag10 IgG levels the control group, but there was no significant difference in the IgG levels between these two subgroups. Spearman correlation analysis further verified the relationship between anti-ApoB100 IgG antibodies and clinical characteristics, but no correlation between anti-Ag1 IgG and clinical characteristics was established either in the patient group or in the control group (Supplementary Table S1). Interestingly, anti-Ag10 IgG antibody was found to be positively correlated with age, HDL, and ApoA level, and a negatively correlated with triglyceride level in control individuals (Table 5). In addition, this antibody showed a positive correlation with LDL in ACS patients (Table 5). These observations are consistent with recent studies [39–46], which suggest that anti-Ag10 IgG antibodies may be protective against atherosclerosis in the control population and that a pathogenic effect of the IgG antibodies on rapid progression of the disease cannot be ruled out in ACS. Nevertheless, our results are in-line with previous studies indicating that anti-oxLDL titers were higher in ACS than controls [20,48] and significantly increased IgG-p210nat concentrations have been found during the first 4 h of the onset of myocardial infarction compared with later time points [49]. The patients with unstable CAD have an underlying pro-inflammatory or immunological state, with evidence of vulnerable plaques that ultimately lead to plaque rupture or rapid disease progression [50]. Immune responses to native ApoB100-derived peptides appear to be dubious, but might reflect non-specific binding of the antibody and an acute unstable status of the body. It is possible that the antibody binding to native ApoB100-derived peptide is generated *in vivo* as a response to a mildly modified oxLDL that plays a pivotal role in developing atherogenesis [49].

There are some limitations of the present study. First, the time point of sampling was 24 h after the onset of ACS. A further prospective study is needed to confirm when the antibodies begin to rise before the acute progression of CAD and if plasma antibody levels are changed rapidly after the onset of ACS; the correlation between anti-ApoB100 IgG levels and major adverse cardiac events should be investigated as well. Second, this is a single-center study and needs replication of the initial finding by independent studies. At last, we have not investigated *in vivo* antigens bound by these antibodies because ApoB molecules are too big to carry out an *in vivo* study of these linear peptides.

In conclusion, circulating IgG for ApoB100-derived peptide antigens may be a useful biomarker of ACS, although anti-ApoB IgG levels were not associated with the coronary artery plaque burden characterized by the coronary Gensini score.

## Abbreviations

ACS: acute coronary syndrome
ApoB100: apolipoprotein B100
AUC: area under the ROC curve
CAD: cardiovascular disease
CI: confidence interval
hAg: human ApoB100-derived peptide antigen
HDL: high-density lipoprotein
HTN: hypertension
LDL: low-density lipoprotein
NSTEACS: non-ST elevation ACS
NSTEMI: non-ST-elevation myocardial infarction
oxLDL: oxidized LDL
ROC: receiver operator characteristic
SBI: specific binding index
STEMI: ST-elevation myocardial infarction
SBP: systolic blood pressure
TG: triglyceride
